# Quality of randomized controlled trials of new generation antidepressants and antipsychotics identified in the China National Knowledge Infrastructure (CNKI): a literature and telephone interview study

**DOI:** 10.1186/s12874-018-0554-2

**Published:** 2018-09-24

**Authors:** Zheng Tong, Fangzhou Li, Yusuke Ogawa, Norio Watanabe, Toshi A. Furukawa

**Affiliations:** 1 Department of Health Promotion and Human Behavior, Kyoto University Graduate School of Medicine / School of Public Health, Yoshida Konoe-cho, Sakyo-ku, Kyoto, Kyoto 606-8501 Japan; 2 Department of Neurology, Kyoto University Graduate School of Medicine, Yoshida Konoe-cho, Sakyo-ku, Kyoto, 606-8501 Kyoto Japan

**Keywords:** Randomized controlled trials, Antidepressive agents, Antipsychotic agents, Risk of bias, Research integrity, China

## Abstract

**Background:**

We are witnessing an exponential increase in the number of randomized controlled trials (RCTs) reported from mainland China. The increase is particularly notable in the field of new generation antidepressants and antipsychotics. Several previous studies have raised doubts regarding their quality. However, the quality of most recent RCTs published in China may have improved.

**Methods:**

We searched RCTs that examined new generation antidepressants and antipsychotics published between 2013 and 2016 in the China National Knowledge Infrastructure (CNKI), the largest database of scientific publications in China. We interviewed the authors of a random subset of the identified references. We assessed the methodological rigor of each study based on the published reports and telephone interviews with the authors using six methodological domains adapted from the Cochrane’s risk of bias tool.

**Results:**

The final sample consisted of 138 studies, for which we interviewed 58 authors; the authors of 51 studies declined the interview, and the authors of 29 studies could not be contacted. The 51 studies with refused interviews were significantly less likely to be reported from university-affiliated hospitals and were less likely to be published in Chinese core journals. Based on the published reports, most of the 58 studies were assessed to be at unclear risk of bias in most methodological domains. After the interview, only 10 studies were assessed to be at low risk of bias for sequence generation and allocation concealment. Assuming that the studies for which the authors declined interviews had an unclear risk, the proportion of RCTs at low risk of bias in both sequence generation and allocation concealment was 9.2% (10/109, 95% confidence interval [CI]: 5.0 to 16.2). The interviews indicated that the studies were at high risk of bias for most of the other domains.

**Conclusion:**

In general, RCTs that evaluate new generation antidepressants or antipsychotics and are indexed in the CNKI continue to be of low quality. When conducting systematic reviews and meta-analyses in this field, it would be wise to include a specialist from China as a coresearcher to help assess the risk of bias in the identified studies.

**Electronic supplementary material:**

The online version of this article (10.1186/s12874-018-0554-2) contains supplementary material, which is available to authorized users.

## Background

Clinical studies from China have been attracting worldwide attention as their publications have increased in recent years in both absolute quantity and relative share in the world [1–3]. We particularly note the exponential surge in studies published in China in the field of psychiatry that examined new generation antidepressants and antipsychotics [4]. For example, the 2014 update of the Cochrane review of aripiprazole for schizophrenia [5] identified 170 new trials, 162 of which had been conducted in China, in addition to the 4 trials in their original 2009 review [6].

However, many Chinese trials can be accessed only through searches in the Chinese databases [4] and have not been adequately integrated into the world medical literature. For example, less than 3% of the published Cochrane reviews had searched major Chinese databases [7], even though they are supposed to conduct a comprehensive search to avoid publication bias [8]. This lack may be due, in part, to the language barrier; however, it is also likely due to the purported low methodological quality of most studies published in China. Reports of clinical trials from mainland China have been considered of low quality for various reasons. First, Wu et al. [9] reported after interviewing 2235 authors by telephone that only 6.8% of published randomized controlled trials (RCTs) between 1995 and 2005 identified in the China National Knowledge Infrastructure (CNKI) adhered to accepted methodology for randomization. They found that 85.6% of Chinese authors did not completely understand the principles of randomization when they claimed that their trials were RCTs [9]. Second, the integrity of Chinese clinical studies has been under serious scrutiny because of a high proportion of studies that have reported ‘statistically significant’ results [10], no to extremely low drop-out rates [11, 12] and insufficient ethical considerations [10, 13]. Moreover, news in the past few years has implicated an alarmingly high proportion of plagiarism, falsification or fabrication in the scientific and clinical literature from China [13–15].

More than ten years have passed since Wu et al.’s epoch-making study [9], and the quality of recent RCTs published in China may have improved in the interim. Cohen et al. [7] recently reported that including the CNKI in searches “may lead to the identification of a large amount of additional clinical evidence”. However, recent investigations into the quality of Chinese clinical trials have been based only on the published reports [1–4, 16, 17], which may not always accurately reflect the actual conduct of the trials because of poor reporting [11, 16, 17]. It is therefore imperative to investigate the quality of RCTs recently published in China by conducting direct interviews with the study authors. This research would provide evidence for users worldwide in their critical appraisal of the currently available evidence and help systematic review authors to decide whether to include the Chinese databases in their searches when they conduct systematic reviews and meta-analyses.

## Methods

This study has been approved by the ethics committee of Kyoto University Graduate School of Medicine (registration number R1019).

### Eligibility criteria

We included studies that reported the following features: 1) Design: randomized controlled trials; 2) Participants: human; 3) Intervention: new generation antidepressants or antipsychotics; 4) Outcome: treatment effect or side-effect for a psychiatric disease or syndrome; 5) Published between 2013 and 2016; 6) Reported in the Chinese language; and 7) Included in the CNKI, which is the largest database of scientific publications from China excluding Taiwan or Hong Kong [18]. We excluded studies when complementary and alternative medicine (e.g., traditional Chinese medicine, acupuncture), psychotherapy or nursing therapy was used as an experimental condition and when full reports could not be retrieved.

### Literature search

We searched the titles and abstracts in the CNKI with the free text words of ‘randomized’, ‘human’ or ‘patient’, keywords for psychiatric diseases or symptoms, and the names of new-generation antidepressants or antipsychotics. The publication year was limited to 2013 through 2016. Additional file 1: Appendix 1 provides the details of the search terms used.

### Selection of studies

We excluded studies without available full text from the results of the preliminary search and then randomly reordered them. We kept including studies while two researchers independently checked their titles and abstracts followed by the full text according to the full eligibility criteria; the telephone interviewer then made every effort to contact the study authors until we reached the prespecified sample size of successful interviews (See below: Sample size calculation). Disagreement with regard to study eligibility was resolved by discussion and, when necessary, consultation with a third researcher.

### Risk of bias assessment

We assessed the risk of bias of the included studies from two viewpoints: first based on the published report only and second after completing the semi-structured telephone interview (See below: Telephone interview) with the study author. The tool for assessing the risk of bias was adapted from the Cochrane’s risk of bias tool [8] and included the following six domains: 1) sequence generation, 2) allocation concealment, 3) blinding of participants, personnel and outcome assessors, and 4) intention-to-treat analysis, in addition to 5) protocol registration and 6) provision of the protocol (only for the telephone interview). Each domain was categorized as having a low risk of bias, a high risk of bias, or an unclear risk of bias. Additional file 1: Appendix 2 provides the operationalized definitions and guidance for the judgments of the risk of bias in each of the six domains. Two researchers who are native speakers of Chinese independently completed all assessments. Disagreement was resolved by discussion and, when necessary, in consultation with a third researcher.

### Telephone interview

We initially used the information provided in the published report to search for the telephone number of the institutions. If this search was unsuccessful, we contacted the journal’s editors, the local health authorities or personal acquaintances for help. The call was typically initiated with the corresponding author; if this approach was unsuccessful, we tried the first author; if unsuccessful, then the second author, and so on, until we exhausted the author list. We often had to call multiple times over several weeks before reaching the author because of the complexity of hospital departments. If we were unable to contact any of the authors from a study even after trying at least three times per week, at different times of the day for at least 4 weeks, we considered this study a report for which the authors could not be contacted. If the authors from one study declared that they were unable or unwilling to answer the interview or if the authors said they were busy three times without providing a further available time, we considered this study a report with interview refusal, with the respective refusal reasons recorded.

The interview was semi-structured and conducted in Chinese by a native speaker of the language (Additional file 1: Appendix 3). We first introduced ourselves and explained the purpose and contents of our study, and obtained an oral consent before proceeding with further interview. We accepted if the author preferred to answer by Email or Wechat (a widely used instant message application in China). We tried to use open questions following the interview guideline to obtain information about the trial’s methodological details and probed if the answer was not clear. We tried to be as non-judgmental as possible during the interview. When permission was granted, we recorded the interview without the names of the authors or institutions. All interviews were recorded and independently assessed by two researchers. Disagreement was resolved by discussion and, when necessary, consultation with a third researcher.

### Outcomes

Our primary outcome was the proportion of RCTs assessed to be at low risk of bias for both sequence generation and allocation concealment based on the interview results (RCTs at low risk of bias in random allocation). We referred to the previous study by Wu et al. [9], considering proper conduct of randomization to be crucial for randomized controlled trials.

Our secondary outcomes included: 1) the proportion of studies at low risk of bias for each domain based on published reports only; and 2) the proportion of studies at low risk of bias for each domain based on the interview results.

### Sample size calculation

We assumed the proportion of RCTs at low risk of bias in random allocation included in our study would be greater than 6.8% (95% confidence interval [CI]: 5.9% to 7.7%), which was reported in the previous study [9]. If we assume 20% to be RCTs at low risk of bias in random allocation, with a sample size of 50 cases who would participate in the telephone interview, the 95% confidence interval (CI) would be 11.2% to 33.2%. If we assume the proportion of RCTs at low risk of bias in random allocation to be 50%, again with a sample size of 50 completed interviews, the 95% CI would be 36.5% to 63.5%. We considered these figures would be helpful in determining whether it is worthwhile to search the CNKI.

### Statistical analysis

We compared the characteristics of the reports that included successful interviews with refused interviews and uncontactable authors in terms of the sample size, statistical significance of the primary outcome, risk of bias based on the published literature, and several quality-related characteristics reported in previous studies, including the hospital’s level where the study was conducted [9] and publication in core journals [19]. Chinese core journals were defined according to the Beijing University Library or China Scientific and Technical Papers and Citations Database. The conventional threshold for statistical significance (*p* < 0.05) was adopted. The proportion of RCTs at low risk of bias in random allocation and the 95% CI were calculated. A sensitivity analysis for the primary outcome was conducted, assuming the reports with refused interviews to be at an unclear or high risk of bias. We used JMP software [20].

## Results

### Characteristics of the selected studies

**Fig. 1:**
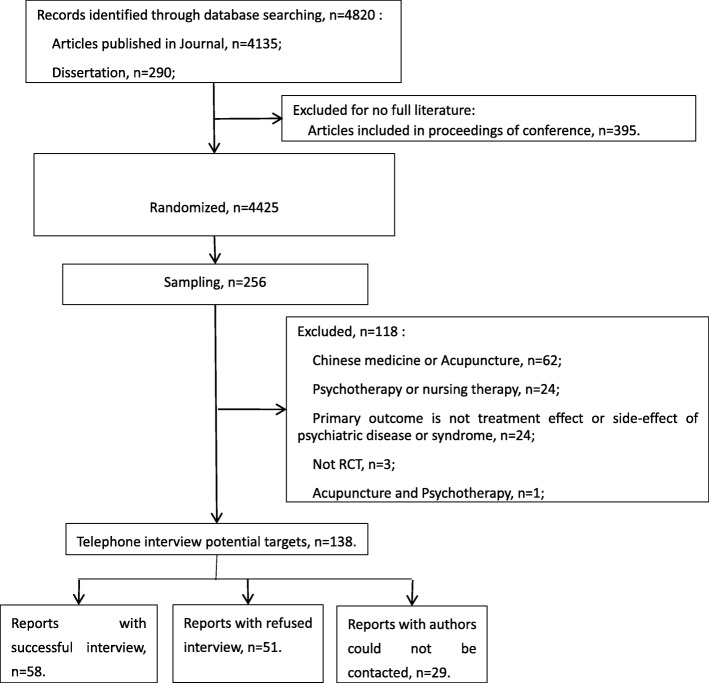
Flow chart of search and selection of studies for interviews

Figure 1 shows the flowchart for the identification and selection of the relevant RCTs. We searched the CNKI on March 8th, 2017 and identified 4820 records. We finally included 138 studies to reach the pre-specified sample size and completed 58 interviews.

Most of the 138 included trials compared different pharmacological treatments and lasted several weeks. The most frequently trialed drugs included olanzapine (*n* = 35), risperidone (*n* = 31), escitalopram (*n* = 25), paroxetine (*n* = 22) and ziprasidone (*n* = 20) among others. All were Phase IV studies. Sixty-two reports (44.9%) had English abstracts. Only 10 trials (7.2%) provided their funding information, all of which were scientific research projects, and all authors of these 10 reports participated in our interview. Twenty-four reports (17.4%) reported approval by ethics committees. Sixty-one reports (44.2%) described obtaining informed consent of the participants.

### Characteristics of reports for which authors could not be contacted or refused the interview

Only 28 reports (20.3%) provided the corresponding authors’ information, including an email address or telephone number. We managed to identify contact information for 131 studies (94.9%). On average, it took two to 3 days to complete one successful interview. Most telephone interviews lasted between five and 8 min. Of the 58 successful interviews, 52 interviews were conducted by telephone, five by email, and one by Wechat.

The authors of 29 studies remained uncontactable despite our efforts. In addition to seven authors for whom we were unable to find contact information, five authors had moved, and their colleagues were unable to provide their new contact information; moreover, for 17 studies, we were only able to locate the numbers of busy hospital lines where the authors were unable to adequately answer even after repeated attempts for 1 month.

The authors of 51 studies were contacted and explicitly refused the interview. Table 1 lists the reasons provided by these authors. Some authors who refused our interview admitted to having used a ghostwriting service to publish a paper or having forged reports in other ways (“Somebody else conducted this study”, “Mistook me (interviewer) as an individual from a ghostwriting service”, or “I made up the data”).

**Table 1:** Reasons provided for refusing an interview

Reasons for refusal	Number
Somebody else conducted this study, and I don’t have his number now.	11
“Busy” for 3 times	11
Too old that I cannot remember	9
I’m not willing to answer / This is not a good time for me.	8
Mistook me (interviewer) as an individual from a ghostwriting company and refused to listen anymore	4
Do not call me anymore.	3
Refused contact through other routes	2
I looked up some similar studies and made the data up.	1
I need my leader’s permission about these things, so I cannot talk more.	1
I only published this paper for my promotion, and it means nothing to you.	1
Total	51

Table 2 compares the study characteristics of the reports with refused interviews and the reports for which the authors could not be contacted with the reports that had successful interviews. The reports with refused interviews were significantly less likely to be conducted in university-affiliated hospitals and were less likely to be published in core journals of the China Scientific and Technical Papers and Citations Database than the reports with authors who agreed to be interviewed.

**Table 2:** Characteristics of reports

	Reports with refused interviews; *n* = 51	Reports for which authors could not be contacted; *n* = 29	Reports with successful interviews; *n* = 58
Sample size per study arm			
Less than 30	14, 27.5%	3, 10.3%	10, 17.2%
30 to 60	32, 62.7%	22, 75.9%	39, 67.2%
60 to 100	3, 5.9%	3, 10.3%	8, 13.8%
More than 100	2, 3.9%	1, 3.4%	1, 1.7%
Primary outcome between groups			
P < 0.05	35, 68.6%,	21, 72.4%	33, 56.9%
*P* ≥ 0.05	16, 31.4%	8, 27.6%	22, 37.9%
Others (multiple outcomes showed different results)	0	0	3, 5.2%
Assessed to be at low risk of bias based on published reports			
1. Sequence generation	7, 13.7%	4, 13.8%	16, 27.6%
2. Allocation concealment	0	0	0
3. Blinding of at least one available object	0	0	2, 3.4%
4. Intention-to-treat analysis	47, 92.2%	25, 86.2%	52, 89.7%
5. Registered protocol	0	0	0
Hospital’s level where the study was conducted			
Hospitals affiliated with medical universities	2, 3.9%*	7, 24.1%	13, 22.4%
Level 3 hospitals (including hospitals affiliated with medical universities)	23, 45.1%	15, 51.7%	27, 46.6%
Level 2 hospitals	24, 47.1%	9, 31.0%	27, 46.6%
Level 1 hospitals	1, 2.0%	0	1, 1.7%
No information	3, 5.9%	5, 17.2%	3, 5.2%
Published in core journal			
Core Journal from list of Beijing University Library (2014 version)	0	1, 3.4%	2, 3.4%
Core Journal from list of China Scientific and Technical Papers and Citations Database (2016 version)	5, 9.8%*	5, 17.2%	21, 36.2%

Comparisons were made between the studies with successful interviews (reference) and the studies for which the authors could not be contacted and the studies for which the authors refused the interview using one-way ANOVA for sample size or Fisher’s exact test as appropriate. *: *p* < 0.05 compared with the reference

### Risk of bias among the 58 studies for which authors were successfully interviewed

**Fig. 2:**
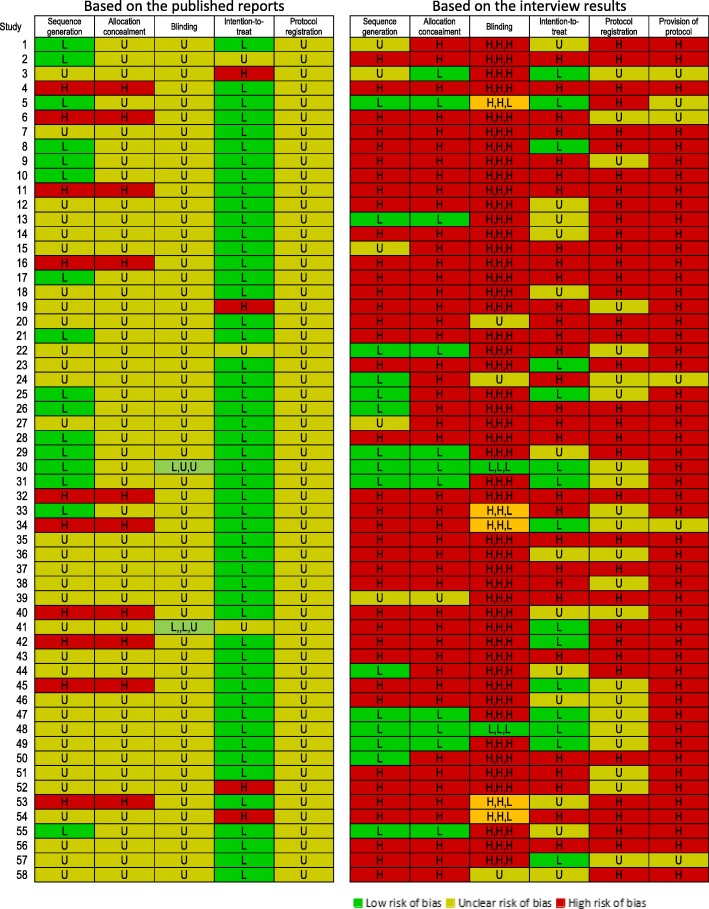
Risk of bias assessment for 58 successfully interviewed reports

Figure 2 shows the risk of bias assessment of the 58 studies for which we obtained interview data. The assessments based only on the published reports were mostly unclear (Fig. 2, left). The assessments substantially changed when the interviews were conducted (Fig. 2, right). The details of the assessments are listed as follows by domain:

1. **Sequence generation**: With regard to the sequence generation as assessed from the publications, 16 reports were rated to be at low risk of bias, using a random number table or other randomization method; 10 reports were assessed to be at high risk of bias, describing the use of alternation depending on the time order of inclusion; and 32 reports were assessed to be at unclear risk of bias due to lack of information to make a judgment.

After the interview, we found that among the 32 reports assessed to be at unclear rsik based on the literature, 20 reports exhibited a high risk of bias, using alternation depending on the time order (*n* = 11), following the clinicians’ prescription (*n* = 6) or other inappropriate methods (*n* = 3). Among the 16 reports originally assessed to be at low risk of bias, eight reports were assessed to be at high rsik, one report was assessed to be unclear, and only 7 reports were assessed to be at low rsik. During the interview, we determined some authors did not completely understand the concept of random allocation because they continued to talk about the inclusion criteria instead of the randomization method when asked about sequence generation.

2. **Allocation concealment**: Allocation concealment was never discussed in the publications. Ten reports were assessed to be at high risk of bias because they explicitly indicated that they used alternation depending on the time order, and their studies were all reported to be conducted in one department of a single hospital.

After the interview, an additional 36 reports were assessed to be at high risk of bias, and only 11 reports were assessed to be at low risk of bias, as the authors claimed that the treatment assignment was managed by personnel separate and independent from the doctors who recruited patients and that the doctors who enrolled the next patient were unaware of the next allocation.

3. **Blinding of participants, personnel and outcome assessors:** Fifty reports did not provide information with respect to blinding in the publications. Among the remaining eight reports, four reports included keywords of ‘double-blinding’ (*n* = 3) or ‘single-blinding’ (*n* = 1) without explaining how and with whom the blinding was conducted or whether the blinding was successful. Two reports noted outcome assessors’ blinding; however, they did not include details to allow judgment. These 56 reports were assessed to be at unclear risk of bias. One report was assessed to be at low risk of bias for blinding participants and personnel by reporting the use of identical-looking capsules, distributing pills by the same time schedule between groups and keeping the related personnel unaware of the allocation. One report was at low rsik for blinding only participants by reporting the same appearance and taste of pills.

After the interview, we determined that the authors of 37 reports admitted to not intending to conduct blinding in studies. Moreover, the authors of nine reports misunderstood blinding for “not telling them that they were in a study” or “not telling the patients which pills they were taking”. The authors of five reports claimed they used outcome assessors from other departments to maintain the blinding of outcome assessors. Only two reports were assessed to be at low risk of bias for blinding of participants, personnel and outcome assessors, using placebos and independent researchers to manage the allocation information blinded from participants, personnel or outcome assessors during the studies. In summary, of the eight reports that claimed some blinding, the interviews indicated that four reports were at high risk of bias.

4. **Intention-to-treat analysis:** Fifty-one reports were assessed to be at low risk of bias with regard to the intention-to-treat analysis, with 40 reports indicating no drop-out at all. Three reports failed to report the number of participants for the outcome and were assessed to be at unclear risk of bias. Four reports were at high risk of bias for reporting unbalanced drop-outs between groups or claiming “exclude all participants who dropped out” as exclusion criteria without reporting the number of drop-outs.

However, according to the interview results, 18 of 40 reports that reported no drop-out in the publication had drop-outs that were simply excluded from the analysis. Altogether, 30 reports were assessed to be at high risk of bias based on the interview results, with 21 authors indicating that drop-outs were excluded from analysis and reporting. The authors of 13 reports indicated that they did not quite remember the drop-outs; thus, their reports were assessed as unclear. In the 15 reports assessed to be at low risk of bias, the drop-out rate reported in the paper was very low; however, the authors provided reasonable explanations, for example, that the participants included in the study were all inpatients, the observational period was short, or the side-effects of the drugs were benign.

5. **Registered protocol:** No published reports cited the trial registration. The authors of 52 reports admitted to no registration, and for the remaining six reports, the authors’ answers were ambiguous.
6. **Provision of the protocol:** There was no description of the study protocol in any of the 58 publications. The authors of 36 reports admitted that they had no protocol or had only simple plans: these studies were rated to be at high risk of bias for protocol. Four authors indicated that their studies were reported as RCTs although they were actually retrospective studies. For the 22 reports for which the authors claimed to have a protocol for their studies, we asked them to send us a copy; however, no authors complied.

Overall, the proportion of RCTs at low risk of bias in random allocation based on the interview data was 17.2% (10/58, 95% CI: 9.6% to 29.0%). Excluding the uncontactable authors but including the authors who explicitly refused the interview in the denominators and assuming them to be at unclear risk of bias, the proportion of RCTs at low risk of bias in random allocation was 9.2% (10/109, 95% CI: 5.0% to 16.2%).

## Discussion

In this study, we assessed the quality of recent RCTs published in China that examined new generation antidepressants and antipsychotics. The sample included a randomly selected subset of relevant RCTs identified in the CNKI, the largest scientific literature database in mainland China, published between 2013 and 2016. The risk of bias assessment was initially based on the published reports and then on telephone interviews with the authors. Of the randomly selected 138 reports, 29 authors could not be contacted, and 51 authors were contacted but refused the interview; we were able to successfully conduct semi-structured interviews with 58 authors (42.0%). The published reports often lacked methodological information and were mostly assessed to be at unclear risk of bias. After the interview, many of the studies originally found to be at unclear risk and some studies at low risk were judged to be at high risk of bias. Based on the telephone interview results, the proportion of RCTs assessed to be at low risk of bias for random sequence generation and allocation concealment was 17.2% (10/58, 95% CI: 9.6% to 29.0%). If we include the studies for which the authors refused the interviews, a plausible estimate for the proportion of RCTs at low risk of bias in random allocation may be 9.2% (10/109, 95% CI: 5.0% to 16.2%). According to the interview results, the other domains of the risk of bias assessment including the blinding of participants, personnel and outcome assessors, the intention-to-treat analysis, the registration of the protocol and the availability of the protocol were mostly assessed to be at high risk of bias. Only two reports were rated at low risk of bias for all domains of random sequence generation, allocation concealment, blinding of participants and personnel, and blinding of outcome assessors. One additional study was rated at low risk of bias for random sequence, allocation concealment, and blinding of outcome assessment.

Our estimate for the proportion of RCTs at low risk of bias in random allocation is similar to that of the previous study, which examined trials published between 1994 and 2005 and found 6.8% (95% CI: 5.9% to 7.7%) to be such RCTs [9]. The quality of the RCTs identified in the CNKI appears to have made little improvement in the intervening 10 or more years. The included studies were mostly small with sample sizes of less than 60 per arm and reported extremely low drop-out rates. The majority of the studies reported positive significant results. We also found that there was no instance of a properly registered protocol, no author provided their protocol to us, and some studies had no protocol at all, all of which clearly affected the quality of the studies. Other research has indicated similar results and found that Chinese authors had a low understanding of protocols [21]. As far as we could ascertain, none of the studies in our sample was registered in a Chinese or international trial registry or was indexed in PubMed.

Fewer than one in five studies reported ethical approval, and less than half reported obtaining informed consent from the participants. Moreover, we found that some authors admitted to falsifying their studies by reporting their retrospective data as RCTs or fabricating their studies from data in the literature or using a ghostwriting service. Some individuals suggest clinical trial fraud is “an open secret” in China [13]. Professor Hu, an expert in biomedical statistics at the Academy of Military Medical Science and the vice-president of the Chinese Society of Biomedical Engineering, also referred to these trends and considered them to be due to a lack of education in clinical study methodology and professional ethics in China [22]. The academic competition of “publish or perish” may also be at play here as the number of publications carries substantial weight in obtaining an advanced degree, job application, promotion and grant application [14].

**Fig. 3:**
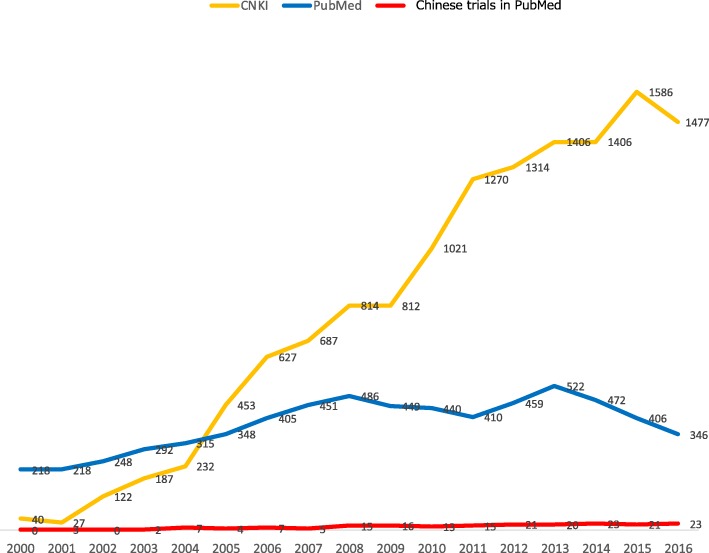
“Randomized” clinical trials of antidepressants and antipsychotics conducted in China, identified in the CNKI (yellow) and PubMed (red) after 2000, superimposed on all such trials identified in PubMed (blue)

Notwithstanding the very low proportion of RCTs at low risk of bias in random allocation identifiable in the CNKI, the sheer volume of clinical trials from mainland China makes them non-ignorable. Figure 3 depicts the numbers of claimed randomized clinical trials of antidepressants and antipsychotics from China in the CNKI (yellow) and PubMed (red). Assuming 10–20% of the trials identified in the CNKI to be at low rsik for both sequence generation and allocation concealment, 100–300 trials can be identified in the CNKI in recent years. If we ignore them, we could be missing one-third to one-half of the relevant trials that researchers have conducted with these agents for psychiatric disorders. However, our experiences in the course of the present study indicate that identifying RCTs at low risk of bias among those from the CNKI can be an onerous task: simple inquiry with the study authors may not be sufficient; ascertaining the pre-published protocol and the source data may be required [13].

There are several limitations to this study. First, we identified only RCTs that examined new generation antidepressants and antipsychotics in the CNKI, and the results may not be generalizable to RCTs from China in other specialties or RCTs identified from China found in other databases. The CNKI lacks an index of recommended journals, such as the Core Journals from the Beijing University Library or China Scientific and Technical Papers and Citations Database, and the results may be different if we limit the search for the relevant Chinese trials to these selected journals. The results would also be different if we assessed trials from China published in international databases, such as PubMed, as higher quality studies from China are more likely to be published in English rather than in Chinese. Second, although we attempted to assess the actual conduct of the studies, the authors’ answers were often ambiguous and remained vague, such as “I don’t remember clearly, but I suppose so.”. We had to rate these instances as being at unclear risk of bias. In some cases, we also had to provide a list of possible answers as hints, and most of the telephone interviews were conducted on the busy hospital office line within a short time, which may have limited the quality of the interview. In the risk of bias assessment for allocation concealment, although there were cases for which we were able to ascertain through successive questions that the allocation was managed by separate and independent personnel and the doctors recruiting the patients were unaware of the next allocation, no researcher reported using a more rigorous central randomization scheme, such as telephone-based or web-based systems. Third, the response rate was 42.0% (58 of 138), which is substantially lower than that of the previous study, 73.1% [9]. The main difference was the refusal rate of 37.0% versus the previous study’s 2.8%. It is possible that the researchers or clinicians who published papers have become more alert to telephone interviews than 10 years ago, thus avoiding answering anything that may be detrimental to them. It remains a plausible conjecture that some authors refuse because they have fabricated their paper as some of the refusal reasons in this study implied. We also identified differences in the quality-related characteristics, which imply that reports with refused interviews have a lower quality than reports with successful interviews. Finally, and most importantly, it cannot be emphasized enough that scientific misconduct occurs in many disciplines and many countries in the world, including Japan, the UK and the USA, as well as many other countries [23–28]. This study focused on recent reports of supposedly randomized trials of new generation antidepressants and antipsychotics in the largest Chinese database, in the current contexts of their exponential increase and their suspected quality.

In contrast, the strengths of the current study may be summarized as follows. This investigation is the only study to conduct telephone interviews of authors to investigate the quality of RCTs recently published in China since the previous study conducted more than 10 years ago [9]. Inadequate reporting of RCTs by Chinese authors makes direct contact with the authors a sine qua non to assess their real quality. We had to expend enormous efforts to contact the authors due first to a lack of corresponding information in the publications and second to some authors’ uncooperative attitudes.

## Conclusion

In conclusion, the RCTs that examined antidepressants and antipsychotics published in the most recent years in China and identified in the CNKI were not only poor overall in reporting but were also often inadequate in their conduct. The clinical implications of the present findings may be as follows: (i) When systematic reviewers search for randomized evidence in Chinese databases, it is imperative that they work in collaboration with Chinese-speaking colleagues to verify the study design, eligibility, and other trial characteristics by contacting the authors and checking their protocols and source data; (ii) Evidence users are advised to exercise appropriate caution in using the systematic reviews that uncritically incorporated trials identified in Chinese databases. Similar interview studies for RCTs reported in other non-English languages are also warranted to adequately gauge the impact of the language bias in the identification of the total body of evidence [29, 30].

With regard to future research to be reported from China, we would like to call for the full registration of trials in the Chinese Clinical Trial Registry (http://www.chictr.org.cn) and accurate and complete reporting of trial protocols and trial results following the respective reporting guidelines [31–33]. Journal editors and peer reviewers should help improve the quality of trials by adopting similar requirements. Journal publishers may also wish to allow trialists to use more space to publish their results, including online supplementary documents as a complementary tool to increase the transparency of reporting. It is important news that the Chinese government is also cognizant of the need for and is determined to invest in, progress in research integrity in China [34, 35]: we look forward to seeing positive changes in the near future.

## Additional file

Additional file 1: Appendix 1 Free text words for searching. Appendix 2 Tool for assessing risk of bias. Appendix 3 Telephone Interview Guideline. (DOCX 28 kb)

## Abbreviations

CI: Confidence interval
CNKI: China National Knowledge Infrastructure
RCT: Randomized controlled trials
