# Supplementary material for: Quality of randomized controlled trials of new generation antidepressants and antipsychotics identified in the China National Knowledge Infrastructure (CNKI): a literature and telephone interview study
Source: BMC Med Res Methodol. 2018 Sep 24;18:96. doi: 10.1186/s12874-018-0554-2 (PMC6154421; doi:10.1186/s12874-018-0554-2)
Supplement: Supplementary file 1 — Appendix 1 Free text words for searching. Appendix 2 Tool for assessing risk of bias. Appendix 3 Telephone Interview Guideline. (DOCX 28 kb) [file 12874_2018_554_MOESM1_ESM.docx]

Additional files for:

Quality of Randomized Controlled Trials of New Generation Antidepressants and Antipsychotics Identified in the China National Knowledge Infrastructure (CNKI): A Literature and Telephone Interview Study

by Tong Z, Li F, Ogawa Y, Watanabe N, and Furukawa TA

# Appendix 1. Free text words for searching

| Category of words | In English | In Chinese |
| --- | --- | --- |
| Study design | ‘randomized’ / ‘randomised’ | ‘随机’ |
| Participants | ‘human’ or ‘patient’ | ‘人’, ‘患者’ |
| Intervention (New generation of antidepressants and antipsychotics) | ‘antidepressants’, ‘agomelatine’, ‘bupropion, citalopram’, ‘desvenlafaxine’, ‘duloxetine, escitalopram’, ‘fluoxetine’, ‘fluvoxamine’, ‘levomilnacipran’, ‘milnacipran’, ‘mirtazapine’, ‘nefazodone’, ‘paroxetine’, ‘reboxetine’, ‘sertraline’, ‘trazodone’, ‘venlafaxine’, ‘vilazodone’, ‘vortioxetine’;  ‘antipsychotics’, ‘aripiprazol’, ‘asenapine’, ‘clozapine’, ‘iloperidone’, ‘lurasidone’, ‘olanzapine’, ‘paliperidone’, ‘quetiapine’, ‘risperidone’, ‘ziprasidone’ | ‘抗抑郁药’, ‘抗抑郁剂’, ‘阿戈美拉汀’, ‘安非拉酮’, ‘丁氨苯丙酮’, ‘西酞普兰’, ‘地文拉法辛’, ‘去甲文拉法辛’, ‘度洛西汀’, ‘依他普仑’, ‘依地普仑’, ‘西酞普兰’, ‘氟西汀’, ‘氟伏沙明’, ‘左旋米那普仑’, ‘米那普仑’, ‘米氮平’, ‘奈法唑酮’, ‘帕罗西汀’, ‘瑞波西汀’, ‘舍曲林’, ‘曲唑酮’, ‘文拉法辛’, ‘万拉法新’, ‘维拉唑酮’, ‘维拉佐酮’, ‘沃替西汀’;  ‘抗精神病药’, ‘阿立哌唑’, ‘阿塞那平’, ‘氯氮平’, ‘伊潘立酮’, ‘伊洛哌酮’, ‘鲁拉西酮’, ‘奥氮平’, ‘帕潘立酮’, ‘帕利哌酮’, ‘奎硫平’, ‘奎的平’, ‘利培酮’, ‘齐拉西酮’, ‘齐哌西酮’ |
| Outcome (Psychiatric diseases or symptoms) | ‘depressive disorder’, ‘depression’, ‘adjustment disorder’, ‘mood disorder’, ‘affective symptoms’, ‘schizophrenia’, ‘psychotic disorders’, ‘mental disorders’, ‘anxiety disorders’ | ‘抑郁’, ‘焦虑’, ‘适应’, ‘情感’, ‘情绪’, ‘精神’, ‘心理’, ‘心境’ |

# Appendix 2. Tool for assessing risk of bias

This tool was employed to assess the risk of bias in the study, based on published reports and telephone interviews. It was adapted from the Cochrane Collaboration’s risk of bias tool. All changes are marked with underline, and the reasons for the changes are listed on the right point by point.

| Criteria | | Reasons for change |
| --- | --- | --- |
| 1. SEQUENCE GENERATION | |  |
| Criteria for the judgment of ‘Low risk’ of bias. | The investigators describe a random component in the sequence generation process, such as:  Referring to a random number table;  Using a computer random number generator;  Coin tossing;  Shuffling cards or envelopes;  Throwing dice;  Drawing of lots;  Minimization*.  *Minimization may be implemented without a random element, and this is considered to be equivalent to being random. | We ascertained through successive questions in telephone interview that treatment assignment was concealed from the doctors who enrolled the patients  The outcome of interest in this study was depression severity whose assessment would be influenced by knowledge of the intervention. We therefore dropped the exceptions originally contained in the Cochrane table.  We found that the drop-outs were extremely low in most included reports. Thus, we specifically inquired about drop-outs in telephone interviews and incorporated this information into the final assessment.  We also operationalized the criteria to judge the influence of unbalanced dropouts in accordance with previous systematic reviews (Furukawa TA, Salanti G, et al (2016) BMJ Open, 6, e010919; Cipriani A, Furukawa TA, et al (2018) Lancet, 391, 1357-1366.)  We often found it difficult to identify the exact primary outcome in the published reports, as most studies reported 3 or more outcomes without explaining the priority. Moreover, we could not find a sample size calculation in most reports, which would reveal the primary outcome.  We therefore judged that the registration of a protocol would constitute an important aspect in judging the quality of studies from the CNKI.  After the assessment of published reports, we found no protocol available in an online register. We therefore added this item as we had hoped that the authors could provide us with the protocol in their own language, even if it was not registered. We reasoned this would be a minimum requirement for a proper RCT. |
| Criteria for the judgment of ‘High risk’ of bias. | The investigators describe a non-random component in the sequence generation process. Usually, the description would involve some systematic, non-random approach, for example:  Sequence generated by odd or even date of birth;  Sequence generated by some rule based on date (or day) of admission;  Sequence generated by some rule based on hospital or clinic record number.  Other non-random approaches happen much less frequently than the previously described systematic approaches and tend to be obvious. They usually involve judgment or some method of non-random categorization of participants, for example:  Allocation by judgment of the clinician;  Allocation by preference of the participant;  Allocation based on the results of a laboratory test or a series of tests;  Allocation by availability of the intervention. |  |
| Criteria for the judgment of ‘Unclear risk’ of bias. | Insufficient information about the sequence generation process to permit judgment of ‘Low risk’ or ‘High risk’. |  |
| 2. ALLOCATION CONCEALMENT | |  |
| Criteria for the judgment of ‘Low risk’ of bias. | Participants and investigators enrolling participants could not foresee assignment because one of the following, or an equivalent method, was used to conceal allocation:  Central allocation (including telephone, web-based and pharmacy-controlled randomization);  Sequentially numbered drug containers of identical appearance;  Sequentially numbered, opaque, sealed envelopes.  For telephone interview assessment:  The treatment assignment was managed by personnel separate and independent from the doctors who recruited patients, and the doctors who enrolled patients were unaware of the next allocation. |  |
| Criteria for the judgment of ‘High risk’ of bias. | Participants or investigators enrolling participants could possibly foresee assignments and thus introduce selection bias, such as allocation based on:  Using an open random allocation schedule (e.g., a list of random numbers);  Assignment envelopes used without appropriate safeguards (e.g., if envelopes were unsealed, non­opaque or not sequentially numbered);  Alternation or rotation;  Date of birth;  Case record number;  Any other explicitly unconcealed procedure. |  |
| Criteria for the judgment of ‘Unclear risk’ of bias. | Insufficient information to permit judgment of ‘Low risk’ or ‘High risk’. This is usually the case if the method of concealment is not described or not described in sufficient detail to allow a definite judgment – for example, if the use of assignment envelopes is described, but it remains unclear whether envelopes were sequentially numbered, opaque and sealed. |  |
| 3. BLINDING OF PARTICIPANTS, PERSONNEL AND OUTCOME ASSESSORS | |  |
| Criteria for the judgment of ‘Low risk’ of bias. | ~~Any one of the following:~~  ~~No blinding or incomplete blinding, but the review authors judge that the outcome is unlikely to be influenced by lack of blinding;~~  Blinding of participants and key study personnel ensured and unlikely that the blinding could have been broken. |  |
| Criteria for the judgment of ‘High risk’ of bias. | Any one of the following:  No blinding or incomplete blinding~~, and the outcome is likely to be influenced by lack of blinding~~;  Blinding of key study participants and personnel attempted, but likely that the blinding could have been broken, and the outcome is likely to be influenced by lack of blinding. |  |
| Criteria for the judgment of ‘Unclear risk’ of bias. | Any one of the following:  Insufficient information to permit judgment of ‘Low risk’ or ‘High risk’;  The study did not address this outcome. |  |
| 4. INTENTION-TO-TREAT ANALYSIS | |  |
| Criteria for the judgment of ‘Low risk’ of bias. | Drop-outs (difference between patients who were randomly assigned at the beginning and patients for whom all follow-up data were available) is less than 20% of all patients who entered the study.  The difference in drop-outs between intervention and control groups is less than 5% in the case of active comparison and 10% in the case of placebo comparison.  For telephone interview assessment:  The literature was judged to be at low risk of bias, and the author explained why the dropout was rare, for example, the patients were all inpatients and therefore easy to follow. |  |
| Criteria for the judgment of ‘High risk’ of bias. | The difference in drop-outs between intervention and control groups is more than 5% in the case of active comparison and 10% in the case of placebo comparison.  An inappropriate imputation method (e.g., LOCF) has been used to impute drop-outs.  For telephone interview assessment:  If there is no reporting about drop-out or claimed no drop-out in the publication, but the author explained dropouts were excluded from the analysis. |  |
| Criteria for the judgment of ‘Unclear risk’ of bias. | Any one of the following:  Insufficient reporting of attrition/exclusions to permit judgment of ‘Low risk’ or ‘High risk’ (e.g., number randomized not stated, no reasons for missing data provided);  The study did not address this outcome. |  |
| 5. PROTOCOL REGISTRATION | |  |
| Criteria for the judgment of ‘Low risk’ of bias. | The study protocol is available on an online register, and the study’s pre-specified primary outcome has been reported in a pre-specified way;  And the reported outcome is the same as in the protocol. |  |
| Criteria for the judgment of ‘High risk’ of bias. | The primary outcome reported in the published report is different from what is registered in the protocol.  For telephone interview assessment:  If the author explained there was no written protocol before conducting the study or the protocol was very simple that it may miss important design information for PICO (participants, intervention, control, outcome), random allocation method, sample size or study period. |  |
| Criteria for the judgment of ‘Unclear risk’ of bias. | Insufficient information to permit judgment of ‘Low risk’ or ‘High risk’. It is likely that the majority of studies will fall into this category. |  |
| 6. PROVISION OF PROTOCOL (For telephone interview assessment only) | |  |
| Criteria for the judgment of ‘Low risk’ of bias. | For telephone interview assessment:  If the author sent us a copy of the protocol or provided us with a link so we could confirm that the reported primary outcome is completely the same as in the protocol. |  |
| Criteria for the judgment of ‘High risk’ of bias. | For telephone interview assessment:  If the author explained there is no written protocol before conducting the study or the protocol was very simple and may miss important design information for ‘PICO’ (identifying the key concepts of participant, intervention group, control group or outcome), random allocation method, sample size or study period. |  |
| Criteria for the judgment of ‘Unclear risk’ of bias. | For telephone interview assessment:  Insufficient information to permit judgment of ‘Low risk’ or ‘High risk’. This is usually the case when the author indicated that he cannot remember the content of the protocol, and in some cases, the author claimed there was a properly prepared protocol but failed to provide it to us. |  |

# Appendix 3. Telephone Interview Guideline

We started the telephone interview with the following six lead questions for the six domains of methodological quality examined.

**Question 1**. I saw that your report mentioned “randomized” (or method of randomization). How did you allocate the patients to treatments? For example, when a new patient was recruited, how did you decide which group to allocate him/her to?

If the author still did not provide information about randomized allocation: We know there are multiple popular ways to allocate participants, for example, some doctors would use alternation by time order, or a random number table. What did you do to allocate your patients?

If the author answered a random number table upon the interviewer’s hint: So how did you operate with this random number table? Who conducted the allocation?

**Question 2**. Was the clinician the one to ascertain that the patient satisfied the eligibility criteria, and also to decide which group the patient would be allocated to? Or was there somebody else responsible for allocating the patient?

**Question 3**. If blinding was not mentioned in literature: Did you try to do blinding or not?

If blinding was mentioned without further detail: When you mentioned double-blinded or blinded, who was blinded? How were they blinded?

If blinding was assessed to be low risk of bias: I noticed your report explained blinding of ___. How about the others involved in your study?

**Question 4**. What did you do when there were dropouts? How did you deal with the missing data?

If no dropout was reported in the report: Were there any patients who could not continue to participate in the study for any reason?

If the dropout rate was low: Could you tell me what you did to maintain this low dropout rate?

**Question 5**. Was this study registered online using the clinical trial registered system?

**Question 6**. Was there a written protocol submitted to the hospital, ethical committee or academic committee? Was it very specific and full of details, or quite simple? Can you tell me what you wrote in the protocol? If possible, could you please send me a copy of your original protocol?
